# High-resolution MRI reveals uteroplacental flow dynamics in a 3D-printed human placental cotyledon model

**DOI:** 10.1126/sciadv.adw9753

**Published:** 2025-08-22

**Authors:** Nirav Barapatre, David Frank, Franz Edler von Koch, Sven Grundmann, Hans-Georg Frank, Martin Bruschewski

**Affiliations:** ^1^ Department of Anatomy II, LMU Munich, Munich 80336, Germany.; ^2^ MRI Flow Lab, University Rostock, Rostock 18059, Germany.; ^3^ Department of Obstetrics and Gynecology, Hospital Dritter Orden, Munich 80638, Germany.

## Abstract

Doppler measurements of the uterine arteries are indirect measures of the uteroplacental blood flow. Given that the intervillous flow cannot be resolved by clinical imaging, theoretical models are used to study the flow dynamics in the intervillous space (IVS). We propose an experimental method to visualize the flow within the IVS of a single placental cotyledon postpartum. At first, a cotyledon is measured by micro-computed tomography imaging. The reconstructed volume is then used to create a near-realistic placenta model. Four variations of arterial inlets are designed to simulate both normal and abnormal flow patterns. A scaled version of the model is printed in three dimensions. Magnetic resonance imaging–based velocity measurements inside the printed model, which is perfused with a Newtonian fluid at two Reynolds numbers, revealed that the flow patterns are primarily influenced by the Reynolds number and the dilation of the arterial inlet. The spiralization of the arterial pathway had only a minimal impact.

## Teaser

The flow patterns inside a born placenta are visualized by experiment to understand the placental blood flow before birth.

## INTRODUCTION

The pregnancy in humans poses a unique challenge on the maternal blood circulation, which undergoes tremendous dynamic changes to continuously accommodate the requirements of a developing fetus in utero (*1*). The fetus, on the other hand, is entirely dependent on the placenta for an efficient exchange of nutrients, metabolites, and gases (*2*). The placental decidual junction represents the basal part of the placental maternal-fetal interface. It is not only the anchoring zone of the placenta but also the crucial region in which maternal blood vessels open into the avascular uteroplacental circulation in the intervillous space (IVS) of the placenta. This region of the placenta is also known as the placental bed. Disorders of this junctional zone can lead to a variety of obstetrical syndromes (*3*). A successful pregnancy, therefore, requires an effective interaction between the maternal and fetal circulations in the exchange zone (the villous tree). For this, a proper uteroplacental blood flow must be established in the IVS around the villi. In addition, the placenta should be well perfused, and the villous fetoplacental circulation must remain uncompromised. Any pathological deviation not only leads to poor fetal growth in utero but also burdens the newborn with an increased risk of chronic diseases (*4*).

The uteroplacental and fetoplacental blood flows are therefore monitored clinically by Doppler ultrasound measurements of the uterine and umbilical vessels, respectively. This provides an indirect assessment of the placental perfusion during routine obstetric examinations. The power Doppler ultrasound method was adapted to measure and understand the placental perfusion directly (*5*). Since then, considerable advances have been made in measuring the blood flow in the IVS by clinical imaging methods like ultrasound (*6*) and magnetic resonance imaging (MRI) (*7*). It has been shown that there is a gestation-dependent change in the inflow through the spiral arteries (*6*). A quantitative MRI study further mapped the net blood velocity and oxygenation in the entire placenta (*7*). Despite the advances made in clinical imaging, it is not yet technically feasible to resolve the flow around the villous tree within a single placental circulatory unit, the cotyledon. Characterizing the flow in a cotyledon is however essential to understand the solute exchange across the placental barrier. This exchange is, in turn, governed by the maternal and fetal hemodynamics and the placental structure. As reviewed by Jensen and Chernyavsky (*8*), many theoretical approaches have been implemented to understand the mechanisms of the feto-maternal exchange. Common to these mathematical models are the assumptions and approximations about either the placental structure or the flow properties or both. The flow patterns are then simulated and visualized by computational methods.

The present study focuses on the uteroplacental flow. It describes an interdisciplinary and experimental approach to visualize the flow within the IVS of a whole placental cotyledon by velocity-sensitive MRI, also known as magnetic resonance velocimetry (*9*). To circumvent the resolution limits of MRI, a cotyledon of a born placenta is measured by high-resolution micro–computed tomography (micro-CT) at first. The reconstructed volume of the cotyledon is then enlarged by a factor of 8 and printed in three dimensions (3D) subsequently. The flow patterns in the 3D-printed model of the cotyledon are lastly visualized by magnetic resonance velocimetry. In this pilot approach, the focus is on the flow patterns in the IVS that are generated as a consequence of changes in the inflow characteristics, like remodeling of the spiral artery and changes in fluid velocity and viscosity. The latter parameters are interrelated and can be described by a single parameter, the Reynolds number. This dimensionless quantity is described as the ratio of inertial to viscous forces of fluid in a given geometry of a conduit. The aim of this study is to investigate by experiment the effect of changes in vessel geometry and Reynolds number on the blood flow pattern in the IVS of a real placental cotyledon.

## RESULTS

**Fig. 1.**
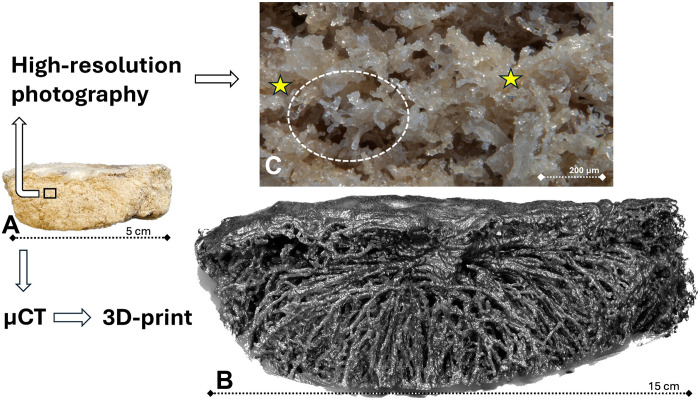
Illustration of alterations linked with the process leading to the creation of a 3D-printed cotyledon. (**A**) Overall image of the cotyledon postfixation, embedding, and air contrasting of the IVS, shown from a side perspective. The cotyledon measures 5 cm in size. (**B**) The 3D-printed cotyledon is magnified threefold and presented from the same side view as the original sample in (A). The alteration in porosity compared to (A) and the prevalence of only large-caliber villi result from the limitation in resolution of the micro-CT imaging technique. The printable portion of the villous structures is also discernible. (**C**) Close-up photomacroscopy displays a section of the villous surface depicted in (A). The IVS contains air, and the method preserves the structure of the villous trees (white oval). Yellow asterisks indicate areas where large villous branches beneath the surface are visible. Smaller branches of villous trees originate from these trunks.

The geometry investigated in this study represents the IVS of a single human placental cotyledon as measured by micro-CT postpartum. The tissue preparation preserves the integrity of the villous tree (Fig. 1). The geometry obtained from the micro-CT data was scaled up initially by a factor of 3 to assess the quality of the 3D-printed model (Fig. 1B). Subsequently, the geometry was scaled up by a factor of 8 to generate a perfusable model for MRI measurements. Upscaling served two purposes: It enhanced the precision of the model’s fabrication and improved the relative spatial resolution achievable with the MRI scanner. This methodological approach provided an experimental access to uteroplacental flow conditions, which had previously been inaccessible through conventional techniques.

During the segmentation process, smaller-scale structures were excluded if their resolution was insufficient for individual identification or when their connectivity to larger structures could not be reliably established. Consequently, some qualitative differences exist between the 3D-printed cotyledonary IVS (Fig. 1B) and the original micro-CT–based geometry (Fig. 1C). From a fluid mechanics perspective, the excluded geometric scales may influence the absolute penetration depth of the jets. Given that the geometry is identical for all investigations, these changes are not expected to bias the outcome of the study, which is comparing the penetration depth for different inlet Reynolds numbers and arterial shapes.

**Fig. 2.**
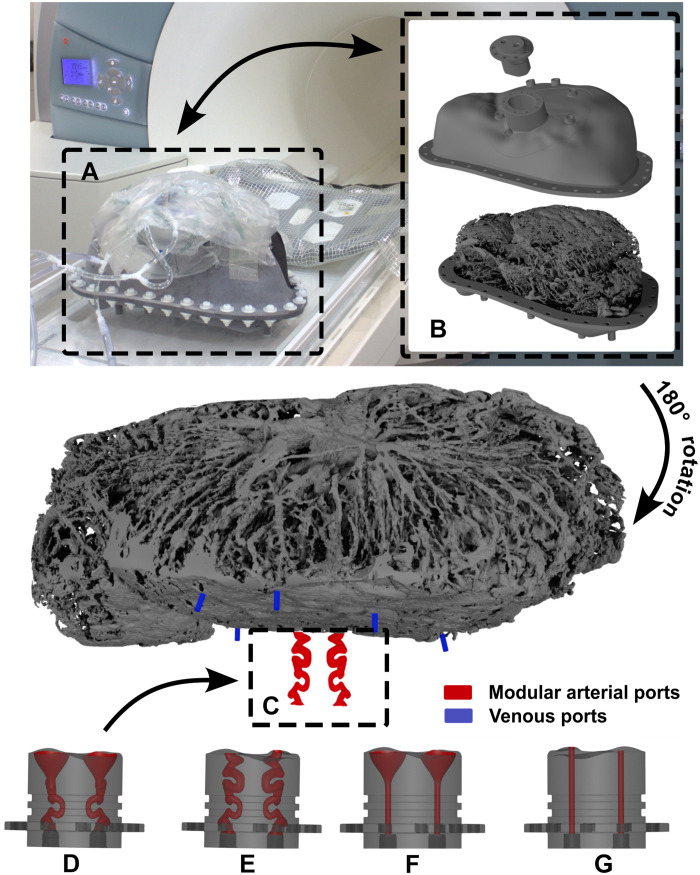
Overview of the placenta phantom. (**A**) Assembled model just before the MRI measurement. (**B**) Exploded view of the model, showing from the top to bottom the inlet module, the lid with venous ports, and the cotyledon structure attached to a supporting structure. For better visualization of the position of arterial inlets (red) and venous outlets (blue), just the cotyledon replica is shown rotated by 180°. The four variations of the arterial ports as shown in (**D**) to (**G**) can be slotted in at position (**C**).

Figure 2 illustrates the complete 3D-printed geometry during the MRI velocity measurement campaign. The modular design allowed for the investigation of four distinct arterial inlet configurations, all subjected to identical boundary conditions. As outlined in Materials and Methods, the Reynolds number similarity was maintained to ensure that the observed fluid mechanics accurately represented physiological blood flow despite the differences in geometric scale and working fluid.

**Fig. 3.**
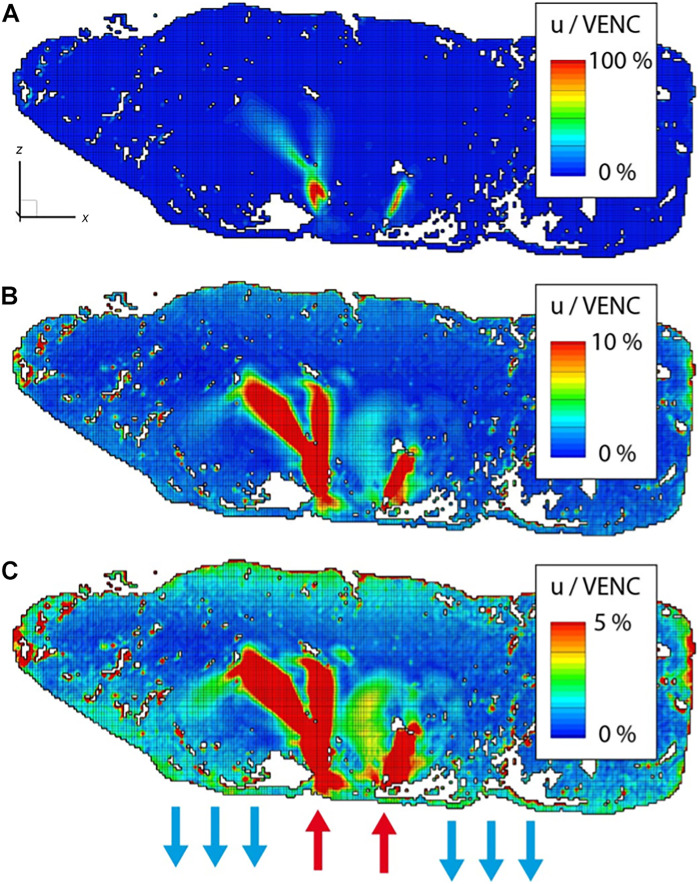
Contour plots of the VENC value. Dynamic range of the 3D MRI velocity measurement demonstrated at a characteristic position. Contour plots show the velocity values up to 100% of the VENC value (**A**), up to 10% of the VENC value (**B**), and up to 5% of the VENC value (**C**). Colored arrows indicated the number of inlets and outlets and their approximate positions.

The precision of the MRI velocity measurements is presented in Fig. 3. The same 2D section of the 3D measurement volume is depicted under three distinct color map limits. The results indicate that the measurement noise was less than 5% of the MRI’s velocity encoding value (VENC), and all major flow phenomena were adequately resolved. Note that the MRI data do not capture the smaller pore scales because of resolution limitations. Despite the partially insufficient resolution, the velocity data accurately reflect the intrinsic fluid velocity within the porous medium as this is a fundamental characteristic of MRI velocity measurements (*10*)

**Fig. 4.**
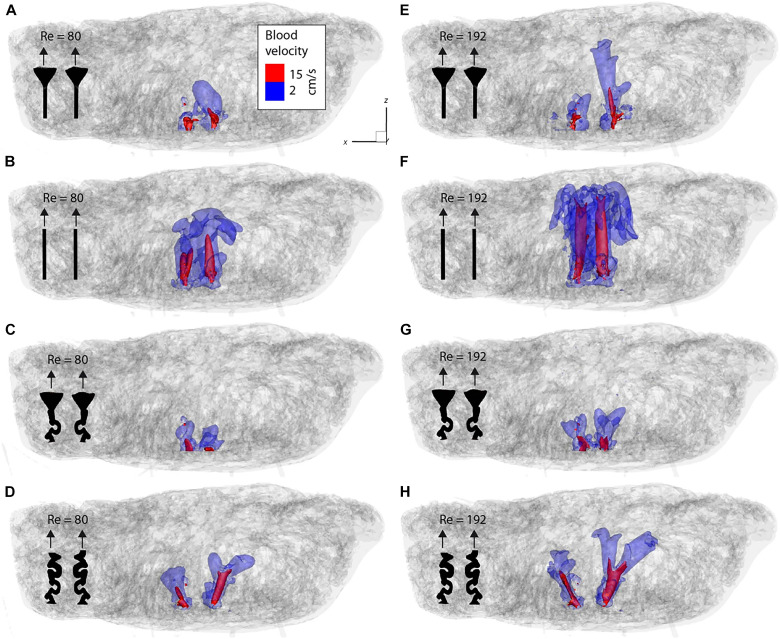
Isovolumes of the fluid velocity. (**A** to **H**) Overview of all eight cases measured with velocity-encoded MRI. The gray geometry is the segmented volume from MRI. The colored surfaces indicate the isovolumes where the blood velocity in the placenta would exceed 2 cm/s (blue) and 15 cm/s (red). The black pictograms indicate the artery geometry.

**Fig. 5.**
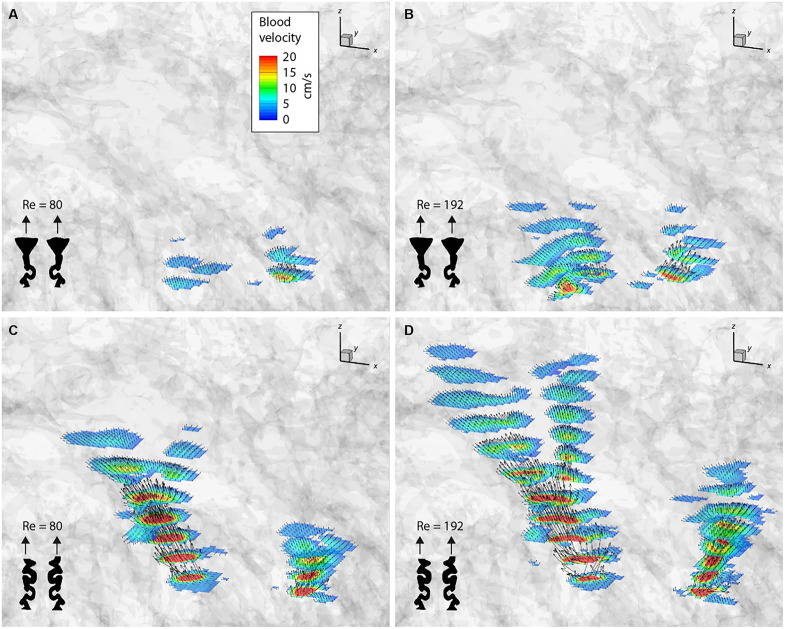
Fluid velocity vectors in the IVS. Distribution of the directions and magnitudes of the velocity vectors for dilated (**A** and **B**) and nondilated (**C** and **D**) spiral arteries at Reynolds numbers of 80 [(A) and (C)] and 172 [(B) and (D)].

For the fluid mechanics analyses depicted in Figures 4 and 5, the measured velocity values were scaled to reflect the corresponding blood flow velocities under physiological conditions using the principles of Reynolds number similarity. A key finding across the eight measured configurations was the variation in penetration depth of the laminar jets. This variation was influenced by two primary factors: the Reynolds number and the distinction between dilated and nondilated arterial openings. Both factors affect the flow state inside the IVS. The local Reynolds numbers based on the pore scale of the IVS depend linearly on the inlet Reynolds number. Dilation, acting as a flow diffuser, reduces the local velocities, similarly leading to lower pore Reynolds numbers. With decreasing pore Reynolds number, the flow eventually transitions from a momentum-driven laminar flow to a Darcian flow (*11*). The Darcian regime describes a flow that is only driven by pressure gradients and is independent of the fluid momentum. At the point of transition, the jet cannot penetrate any farther and the fluid turns toward the venous ports because of the low pressure at these outlets. In effect, a jet with high inlet Reynolds number and a nondilated arterial port can penetrate farther into the IVS because the flow remains momentum-driven for a longer distance.

Spiral inlet conditions demonstrated shallower penetration depths compared to straight inlets under identical Reynolds numbers. This can be attributed to differences in jet angles and the heterogeneous structure of the porous IVS. The shape of the jet velocity isosurfaces suggests that the angled jets encounter more obstructions from larger structures along their paths, thereby reducing their penetration depth relative to the straight inlet configurations.

Figures 4 and 5 also reveal a notable asymmetry in the velocity profiles, with one jet penetrating deeper than the other. This asymmetry cannot be attributed to uneven inlet conditions, as 2D velocity measurements confirmed symmetrical inlet flow. A plausible explanation for this phenomenon is fluid entrainment, where the surrounding fluid is drawn into both jets, creating a low-pressure region between them. This interaction directs one jet toward the adjacent jet, causing the two to merge into a larger jet. Supporting this hypothesis is the observation that the jets appear angled toward each other, even though the straight inlet tubes are geometrically parallel and identical in both the dilated and nondilated configurations.

## DISCUSSION

This study presents an experimental approach to study the intervillous flow at the level of a single placental circulatory unit. The approach involved high-resolution micro-CT imaging of a whole human placental cotyledon, followed by the processing of the imaging data to create a 3D-printable mesh. This mesh was used to construct a scaled, perfusable model of the cotyledon with arterial inlets and venous outlets. Subsequently, the cotyledon model was printed in 3D. The 3D-printed model was lastly perfused with a model fluid of defined Reynolds number, and the flow patterns were visualized and quantified by MRI velocity measurements.

The primary aim of this study was to portray realistic flow scenarios of the placental blood. Furthermore, the study examined the relative significance of two general morphological characteristics of the arterial supply to the IVS. Physiologically, the arteries exhibit a spiral structure with widened openings into the IVS. This study aimed to determine the influence of these two morphological components (spiralization of the arteries and size of the arterial openings) on observed flow patterns within the IVS. Our results indicate that the critical morphological features are the dilation of the arterial opening and the inlet Reynolds number. The spiralization of the artery has a minor impact on the formation of jets within the IVS. Although the contribution of spiralization is minor, it may still be biologically relevant. Conversely, there are ongoing discussions regarding other primary functions of spiralization, such as their role as a reserve for resistance (*12*). Given that spiralization itself is already present in the endometrium before implantation, there might also be endometrium-related functional features of spiralization of the endometrial arteries.

The spiral arteries used in the model were reconstructed from an actual spiral artery that was found by chance attached to the basal plate of a delivered placenta. So, the inlet geometry resembles the in vivo situation. The straight arterial inlets, which are highly improbable to occur naturally, were only constructed to separate the individual contributions of terminal dilation and spiralization. The results clearly show that the formation of jets in the IVS is determined primarily by the terminal dilation. The occurrence of jets had also been predicted by in silico modeling in small regions of the intervillous circulation (*13*, *14*). Roth *et al.* (*14*) used a limited flow area reconstructed from serial microscopic images, whereas Burton *et al.* (*13*) relied on the Hagen-Poiseuille equation for flow calculations and on assumptions derived from the literature. Overall, these data support the findings of the current study regarding the presence of jets in the intervillous area of the human placenta.

Resolving the IVS flow is essential because a suboptimal flow can affect the villous growth. This, in turn, affects the fetal growth. So far, the IVS flow could be studied in vivo only in nonhuman primates with hemochorial placentation by cineradiography (*15*). The jets observed in the present study corroborate these early findings as well. Such cineradiographic studies are not possible in humans because of obvious ethical reasons. Therefore, power Doppler ultrasound methods have been developed to visualize the placental (*5*) and intervillous blood flows (*16*). Notable progress has been made in visualizing the jet formation and quantifying the flow velocity in the IVS (*17*). However, quantifying the flow patterns in a placental cotyledon is technically still not possible.

Theoretical approaches are, therefore, the methods of choice when studying the gas and metabolic exchange at the level of a single cotyledon. From the opening of the spiral artery to the venous drainage, each placental structure that a quantum of blood interacts with is modeled on the basis of assumptions with a varying degree of complexity [see review (*8*)]. As described in this review, the structure of the villous tree or the IVS influences the intervillous flow considerably. The theoretical models either assumed a homogeneous porous medium (*18*) or involved complex assumptions about the spiral artery dimensions and the villous density across different gestational time points (*19*). The heterogeneous and irregular shape of the IVS was shown to have an impact on the oxygen uptake by the villi (*20*). Therefore, it is essential to model with in vivo–like placental geometries. Burton *et al.* (*13*) computed the flow velocity only in the villous-free central cavity for dilated and nondilated arteries. Our results show similar blood velocities in the central parts of the jets in the villous-free region of the central cavity (Figs. 4 and 5). These jets are eventually decelerated substantially by the villous tissue before being drained through the venous ports (Fig. 3). The entire cotyledon measured ~5.0 cm by 3.0 cm by 2.5 cm (37.5 ml). Hence, it represents the largest continuous area of human IVS ever used for rheological analysis.

The experimental model used in this study further holds promise for advancing the morphological 3D representation to more intricate and precise configurations. This approach can be expanded to encompass pathological conditions of the villous tree, such as placentas affected by fetal growth retardation or gestational diabetes mellitus. Another avenue for enhancing this innovative tool for analyzing placental function lies in the investigation of flow-dependent properties, such as the distribution and transfer of oxygen within the IVS. Imaging techniques, which offer a higher resolution, such as those using synchrotron radiation, can improve the resolution of porous spaces.

Aside from the physiological dilation of arterial openings, the most consequential influence on uteroplacental flow was found to be that of Reynolds number. This dimensionless factor quantifies the ratio of inertial forces to viscous forces in a flow. It correlates directly with the volume flow rate entering the placenta and inversely with the viscosity of the blood. If the vascular diameters remain unchanged, then the Reynolds number depends only on the viscosity of the blood and its flow rate. Both these parameters are individual-specific and vary considerably during the course of the pregnancy. Our findings suggest that high-velocity jets in the IVS could occur despite physiological arterial remodeling.

Until now, the Reynolds number has been largely overlooked in the analysis of the uteroplacental flow. The experimental findings of this study underscore its importance and reveal that the Reynolds number cannot be disregarded in investigations of the normal or pathological flow in the uteroplacental circulation. For the Reynolds numbers used in this study, a laminar flow can safely be assumed. Our observations further reveal that for Reynolds number lower than 80, the viscous forces would have dominated and created stagnant zones in the IVS where the fluid would be nearly stationary, and no flow would occur. It is not known how Reynolds numbers vary in vivo. The independent placental contractions, as observed by Dellschaft *et al.* (*7*), could counteract against such stationary states in cases of lower Reynolds number.

Nevertheless, there are certain limitations that must be considered while interpreting the findings of the present study. Given that the cotyledons were fixed in formaldehyde by immersion, tissue shrinkage might have led to alterations in the pore size and the shape of the IVS. So far, the best results for tissue preservation can be achieved by ex vivo dual perfusion of cotyledon, as described by Schneider *et al.* (*21*). Distortion of the IVS can still not be ruled out even in this method.

Furthermore, the resolution limit of the imaging modality, together with the structural losses during 3D printing, has led to a model that has a lower density of terminal villi. Hence, the dampening effects of this part of the villous tree are not reflected in the MR measurements. Also, the use of a Newtonian fluid does not account for the possible, substantial nonlinear effects in the narrow pores within the IVS. The absolute penetration depth of the jets in vivo might be lower than measured in this study. Given that the comparisons are made among different inflow conditions and not among different morphologies of the villous tree, this aspect does not play a critical role in this study.

Last, the effects of venous clearance, placental contractions, as measured by Dellschaft *et al.* (*7*), and uterine contractions have not been considered. This experimental model, however, offers a solid platform, which can be modified, extended, and developed further to include various aspects that were not considered in this study. The loss of terminal parts of the villous tree, for example, can be compensated by growing them in silico on the actual segmented tree before printing in 3D. The properties of the fluid can be varied, and the cotyledon can be printed using a flexible material in the future.

## MATERIALS AND METHODS

All methods and procedures were performed in accordance with relevant guidelines and regulations. The ethics board of Ludwig Maximilians University of Munich (LMU Munich) has approved all investigations under numbers 084-11 and 478-12. The placentas were collected at the Department of Obstetrics and Gynecology of the hospital “Dritter Orden,” Munich, Germany, after an informed consent of the mother was taken.

Placental cotyledons were identified by inspection of the basal plate of a freshly delivered nonpathological placenta. A single cotyledon was dissected, rinsed in a 0.9% NaCl solution, and incubated in a lysis buffer solution for 1 hour to remove the blood cells from the IVS. Subsequently, it was fixed and embedded in paraffin according to standard laboratory protocols.

The paraffin-embedded cotyledon was processed further as per the previously published protocol to replace the paraffin by air selectively from the IVS (*22*). Briefly, the paraffin block containing the cotyledon was placed on a pile of absorbent papers in a beaker. The beaker was kept in a heating oven at 66°C for 3 days. The orientation of the cotyledon was changed twice a day to let paraffin flow out selectively and completely from the IVS. The cotyledon was subsequently allowed to cool at room temperature.

### Micro-CT

The whole cotyledon was then measured by a GE Phoenix V|tome|x s 240 with a nominal isotropic voxel size of 50 μm. With X-AID (MITOS GmbH, Munich), 1601 x-ray projection images were reconstructed into a 3D volume, whereas Fiji/ImageJ was used for image processing and analysis.

Only branch generations that could be identified in the micro-CT images were segmented using the 3D Weka Segmentation algorithm of Fiji/ImageJ. Peripheral branches of the villous trees remained unresolved and, hence, were removed from the image stack. Before 3D printing, the stack was processed further with Blender (Blender Foundation, Amsterdam, the Netherlands), as described below.

### Design of the study to measure intervillous flow

The functional model used for the MRI experiments is based on the cotyledon mesh. The system is simplified to allow for controlled and repeatable measurement conditions while maintaining the most important fluid dynamic features. All structures of the functional model are rigid, and the fluid flow is stationary. The cotyledon replica is enclosed in a chamber with two inlets mimicking arteries and six outlets mimicking the draining veins. The parameters investigated are the Reynolds number and the morphology of the artery. The Reynolds number describes the ratio between inertial and viscous forces and is defined here as

$$\text{Re}=\frac{U_{\text{Artery}}D_{\text{Artery}}}{v}$$

with $U_{\text{Artery}}\;\text{and}\;D_{\text{Artery}}$ being the mean velocity and characteristic diameter of the artery, respectively, and *v* being the fluid’s kinematic viscosity. The Reynolds numbers examined in this study are 80 and 192, representing peak blood flow conditions. It is important to note that peak Reynolds numbers are influenced by vascular diameter, blood viscosity, and flow rate, all of which are highly individual-specific and vary considerably throughout pregnancy. Nevertheless, the selected Reynolds number range aligns closely with values reported in the literature. Burton *et al.* (*13*) documented maximum Reynolds numbers between 20 and 80 based on the arterial radius, which corresponds to a range of 40 to 160 using the definition applied in this study. Mekler *et al.* (*20*) estimated a range of 60 to 180. Many other studies, however, lack sufficient data to precisely determine the investigated range with high certainty.

Four variants of the artery’s morphology were created to study the effect of two distinct anatomical features: the effect of spiral course and the effect of terminal dilation at the outlet of the arteries. The four geometries can be described as follows:

1) A simple cylindrical course with a diameter of 0.4 mm without terminal dilation and placed orthogonal to the cotyledon according to (*13*).

2) A cylindrical course (same as in variant 1) with a conical expansion at the artery’s outlet with a diameter ratio of 6.25. This variant is similar to the description in (*13*) and to the depictions in (*23*).

3) An actual, reconstructed spiral artery that was used in (*14*) with a mean hydraulic diameter of 0.4 mm without terminal dilation.

4) A reconstructed spiral artery (same as in variant 3) with a designed conical expansion at the artery’s outlet (same as in variant 2).

### 3D printing of the scaled cotyledon

The functional model was uniformly scaled by a factor of 8 to allow for a better relative measurement resolution in the MRI experiments. Dynamic similarity between in vitro and in vivo flow conditions was ensured by adjusting the fluid viscosity and flow rate to match the Reynolds number.

The cotyledon mesh for the model was created from the micro-CT data via manual segmentation. Using the software Blender (Blender Foundation, Amsterdam, the Netherlands), imaging artifacts like free-floating structures were removed and the final mesh was defined via an image threshold. The convex hull of the cotyledon was extracted to define the IVS. The hull was then smoothed to mimic in vivo conditions. Next, the cotyledon mesh was cut to shape by the smoothed hull to ensure a close fit of ~0.5 mm within the IVS. This would allow for manufacturing tolerances of ~0.3 mm. A looser fit would risk flow being diverted into this gap, which we aimed to minimize as much as possible. The coordinates of the two arteries were identified by following the central cavity in the micro-CT images toward the basal plate. The coordinates for six veins were identified similarly by locating openings in the periphery of the basal plate.

With all the anatomical shapes and coordinates defined, the hull was split into two parts, which could be assembled later. After adding a wall thickness, the hull section around the two arteries was cut out to facilitate a modular setup. Four artery modules representing the four variants of the arterial morphology were constructed as described earlier. The modular setup ensures identical conditions for measurements with different arterial inlets. Last, a flange connection between the two parts, hose nozzles, and a supporting plate were added (see Fig. 2). The measurement setup was designed in a way that the basal plate, and hence the arterial and venous ports, were facing upward.

The final model consists of three main parts: the supporting plate merged with the chorion hull of the cotyledon replica, a matching lid over the basal plate with venous ports and a slot for the artery module, and four exchangeable artery modules. The chorion hull, supporting plate, and lid were manufactured from polyamide 12 using multijet fusion, while the smaller artery modules were manufactured from resin using stereolithography.

The orientation of the model was chosen for practical reasons, with all access ports positioned on the top of the setup. It does not affect the flow in the IVS because the buoyancy effects are absent as a result of homogeneous fluid density and isothermal conditions.

### Velocity-sensitive MRI measurements

Two different water-glycerin mixtures were used for the experiments, enabling the study of the two different Reynolds numbers with the same setup and measurement sequence. To reduce the relaxation times of the mixture, a small concentration of copper sulfate (1 g/liter) was added (*24*, *25*). Two Newtonian fluids were produced with different material properties. The density and kinematic viscosity of the two samples were measured before the experiments. The first sample has a density of 1189 kg/m^3^ and a kinematic viscosity of 24.617 mm^2^/s, while the second sample has a density of 1158 kg/m^3^ and a kinematic viscosity of 10.235 mm^2^/s. The flow loop comprised a 25-liter tank, an inductive flow meter SM4020 (ifm electronic GmbH, Essen, Germany), and a gear pump, which propelled a steady flow through a 10-mm polyvinyl chloride tube. The main tubes were split into eight equal-length tubes, each connecting to the respective arterial and venous ports of the model. The inductive flow meter measured both the flow rate and temperature with total uncertainties of 15 ml/min and 2.5 K, respectively.

The MRI measurements were performed on a 3-T magnetic resonance imaging system TimTRIO (Siemens Healthineers, Erlangen, Germany) within MRI Flow Lab Rostock (*9*). Using a custom velocity-sensitive sequence, a three-component mean velocity field was acquired with a resolution of 1.5 mm^3^ of the entire model (*26*). Assuming dynamic similarity and taking into account the scaling factor of 8, the measurement resolution is equivalent to 188 μm in the real cotyledon sample. A steady-state flow rate of 594 ml/min and a constant temperature of 20°C were maintained throughout the experiments, resulting in a bulk inlet velocity of 0.615 m/s in both 3.2-mm inlet channels. A low VENC of 0.138 m/s was selected for the MRI measurements to ensure a good overall measurement precision within the acquired velocity data. This overall uncertainty was further improved by repeating each measurement twice and averaging the results. In the end, a total dataset of eight 3D3C (three-dimensional, three-vector-component) mean velocity fields was obtained for four different inlet geometries and two Reynolds numbers of 80 and 192.
